# CoRegNet: reconstruction and integrated analysis of co-regulatory networks

**DOI:** 10.1093/bioinformatics/btv305

**Published:** 2015-05-14

**Authors:** Rémy Nicolle, François Radvanyi, Mohamed Elati

**Affiliations:** ^1^iSSB, CNRS, University of Evry, Genopole, 91030 Evry Cedex, France, ^2^Institut Curie, PSL Research University, 75248 Cedex 05, France and ^2^CNRS UMR144, 75248 Cedex 05, France

## Abstract

CoRegNet is an R/Bioconductor package to analyze large-scale transcriptomic data by highlighting sets of co-regulators. Based on a transcriptomic dataset, CoRegNet can be used to: reconstruct a large-scale co-regulatory network, integrate regulation evidences such as transcription factor binding sites and ChIP data, estimate sample-specific regulator activity, identify cooperative transcription factors and analyze the sample-specific combinations of active regulators through an interactive visualization tool. In this study CoRegNet was used to identify driver regulators of bladder cancer.

**Availability:** CoRegNet is available at http://bioconductor.org/packages/CoRegNet

**Contact:** remy.nicolle@issb.genopole.fr or mohamed.elati@issb.genopole.fr

**Supplementary information:** Supplementary data are available at *Bioinformatics* online.

## 1 Introduction

Recent advances in genomics enabled the profiling of thousands of tumors by large consortia and individual laboratories. While the amount of data holds great promise for our understanding of tumorigenesis, these datasets necessitate efficient methodologies to extract valuable knowledge from them.

Transcriptomics is the most commonly available type of tumor large-scale data. The transcriptome reflects the genetic, epigenetic and environmental states of a tumor tissue and determines a great extent the phenotype of cells. Therefore, one of the first step towards the construction of a mechanistic model underlying cancer is the identification of the sets of transcription factors (TF) that actively maintain a malignant phenotype. This requires methodologies to model the tissue specificity of gene regulation by inferring trustful context-specific networks. More importantly, these models must take into account the complexity of mammalian gene regulation often involving the coordinated action of several TF (Panne, 2008).

To identify tumor-driving active regulatory circuits, we propose a Bioconductor (Huber *et al*., 2015) package named CoRegNet to (i) reconstruct a large-scale co-regulatory network from gene expression data and by integrating additional regulatory evidences such as TF Binding site and ChIP data, (ii) estimate the activity of each TF of the network in any given sample, (iii) predict sets of cooperative TF and (iv) identify sample-specific combination of active and driver TF using an interactive visualization tool integrating genomic aberrations. The proposed methods can be used as independent modules with alternative inputs such as networks inferred by other methods, experimentally defined networks or a different transcriptomic data for TF activity prediction and visualization (e.g. cell lines of same tissue). The following sections outline the functions of the CoRegNet package and its application to the characterization of the driver regulators of bladder cancer subtypes.

## 2 The CoRegNet application

**Fig. 1.**
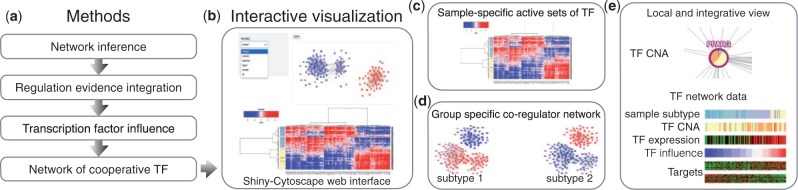
Analysis using the CoRegNet package. **(a)** A set of methods can be used to construct a network of cooperative TF from transcriptomic data using the h-Licorn algorithm and by integrating regulatory evidences. **(b)** A Shiny/Cytoscape web application is available to visually analyze the network and the datasets. **(c)** A dynamic heatmap shows the influence of all or only a selection of TF in all samples. **(d)** The view of the co-regulation network reflects the activity of each TF in the selected samples or sample subtype. **(e)** Copy number aberration (CNA) of TF can be integrated and will first display as a pie graph showing the proportion of each alteration status in either all samples or in the selected subtype. The selection of a single TF will display a multi-layer heatmap to visualize the relationship between sample phenotype and TF expression, activity and CNA

Each following sections are detailed in the Supplementary Information file and the workflow is illustrated in Figure 1.

### 2.1 Regulatory network inference and refinement

To reconstruct a large-scale regulatory network from gene expression data, the CoRegNet package implements the h-Licorn algorithm (Chebil *et* *al.*, 2014; Elati *et* *al.*, 2007). Compared to current methods, h-Licorn focuses on the identification of cooperative regulators of genes. It was proven to have comparable TF-gene pairs prediction performance with state of the art methods in synthetic and Human datasets (Chebil *et* *al.*, 2014) and to retrieve more plausible cooperative TF pairs in yeast (Elati *et al*., 2007, Lai *et al*., 2014) and Human datasets (see Supplementary Information). To obtain a trustful network, regulatory and co-regulatory interactions can be integrated as additional evidences. These can include any type of TF–gene interaction data to support regulatory interactions (e.g. ChIP-seq, TF binding site) and TF–TF interaction data to support cooperative interactions (e.g. protein interactions). The inferred network can then be refined by selecting a subset of the network that is enriched in these external regulatory and co-regulatory evidences using an integrative selection algorithm (Marbach *et* *al.*, 2012).

### 2.2 Transcription factor influence

CoRegNet implements a function to estimate the activity of a TF in a given sample. This is based on a measure of transcriptional *influence*, which was shown to provide a transformed view of the transcriptome in which classification algorithms are more robust (Nicolle *et* *al.*, 2012). Based on a comparison of the expression of the activated and the repressed targets of a regulator, the *influence* is computed in a sample-specific manner (details in Supplementary Information). Robustness of the measure was tested by correlating for each TF the *influence* using the original network and using a partially permuted version of the network with increasing levels of noise. The same was done by correlating the *influence* on the sub-parts of the network that are validated by regulatory evidences. In all comparisons, the *influence* was significantly more robust and consistent with the validated network (e.g. 97% correlation with 20% of noise, 96% for the ChIP validated network) than the other measures of TF activity that were tested (e.g. 75% with 20% of noise and 72% ChIP validation for network component analysis, details and reference in Supplementary Information). Furthermore, the *influence* predicts well the activity of a TF in samples in which it was activated at the protein level by a chemical agonist (details in Supplementary Information).

### 2.3 Constructing a network of cooperative TF

To model the active transcriptional programs, a co-regulation network is built by setting an edge between two significantly cooperative TF (details in Supplementary Information). The relevance of using the h-Licorn algorithm to directly infer a cooperative network is shown by the higher enrichment of the predicted co-regulators in experimentally validated and independently predicted protein interactions (AUPR: 14% for CoRegNet, 6% max. among the four tested algorithms, see Supplementary Information).

### 2.4 Integrative visualization of transcriptional activity

Transcriptomes are summarized by the *influence* of the regulators on the expression of their target genes in the analyzed samples. This abstraction of the transcriptomes through TF activity reduces the number of features thereby simplifying the visualization of the dataset. Moreover, the co-regulation network unravels the combination of TF at work in the studied samples. Thus, an interactive visualization tool is embedded in the CoRegNet package to analyze several layers of information through the sets of active co-regulators. The co-regulation network is accessible through a Cytoscape (Shannon *et al.*, 2003) widget with functionalities to display sets of active co-regulators in particular samples or subtypes (examples in Fig. 1 and in Supplementary Information).

### 2.5 Implementation and availability

CoRegNet is an R package implementing a Shiny (Winston *et* *al.*, 2015) and Cytoscape javascript applet for visualization. The network inference method is implemented in C and can be parallelized. The package is available as a Supplementary File or through the Bioconductor repository.

## 3 Case study

The CoRegNet package was used to analyze a set of bladder cancer samples for which both transcriptome and genomic alterations were available (Biton *et al.*, 2014, Rebouissou *et al.*, 2014). The inferred network was used to estimate the influence of regulators and the visualization tool to identify active sets of master regulators for each bladder cancer subtype. In line with previous studies (Biton *et* *al.*, 2014; Choi *et* *al.*, 2014), PPAR*γ* was found to be the most active TF in the luminal bladder cancer TCGA subgroup I. Association with genomic alteration suggesting PPAR*γ* to be a major driver of these tumors (see Supplementary Information), which has been recently validated experimentally (Biton *et* *al.*, 2014). Moreover, FOXA1, an effector of PPAR*γ* (Varley *et* *al.*, 2008) and a co-factor of GATA3 in luminal breast cancer (Kong *et* *al.*, 2011), is a significant co-regulator of both GATA3 and PPAR*γ* in the inferred network.

## Funding

This work is supported by the INCa (French National Cancer Institute) through the INCa_2960 [PLBIO10] project and the European Union/Framework Programme 7/2009 (“SYSCILIA” consortium, grant 241955). R.N. was supported by a fellowship from the French Ministry of Education and Research. Funding for open access charge: SYSCILIA.

*Conflict of Interest*: none declared.

## Supplementary Material

Supplementary Data
