# Supplementary material for: CoRegNet: reconstruction and integrated analysis of co-regulatory networks
Source: Bioinformatics. 2015 May 14;31(18):3066–8. doi: 10.1093/bioinformatics/btv305 (PMC4565029; doi:10.1093/bioinformatics/btv305)
Supplement: Supplementary Data [file supp_31_18_3066__index.html]

CoRegNet: reconstruction and integrated analysis of co-regulatory networks — CoRegNet: reconstruction and integrated analysis of co-regulatory networks — Supplementary Data 

# CoRegNet: reconstruction and integrated analysis of co-regulatory networks

## Supplementary Data

files

- Supplementary Data - pdf file
- Supplementary Data - gz file
