# Supplementary material for: CoRegNet: reconstruction and integrated analysis of co-regulatory networks
Source: Bioinformatics. 2015 May 14;31(18):3066–8. doi: 10.1093/bioinformatics/btv305 (PMC4565029; doi:10.1093/bioinformatics/btv305)
Supplement: Supplementary Data [file supp_btv305_SupplementaryInformation.pdf]

Supplementary Information
CoRegNet : Reconstruction and integrated analysis of Co-Regulatory Networks

Rémy Nicolle and François Radvanyi and Mohamed Elati

April 17, 2015

1 Comparison to state of the art methods

Functionality comparison

In order to compare the functionalities of CoRegNet to other biological network softwares, a table listing the functionallities of several frequently used softwares is given in table 1. This table is focused on transcriptional network analysis in the context of cancer genomics.

Co-regulator inference

In order to verify the proposed algorithm, a network of cooperative TF is reconstructed from the large-scale transcriptional networks inferred by several algorithms. These included : ARACNE [Margolin et al., 2006], CLR [Faith et al., 2007], WGCNA [Langfelder and Horvath, 2008] and GENIE3 [Huynh-Thu et al., 2010]. Based on the large-scale inferred bi-partite network, containing only edges from TF to other genes, a network of cooperative regulators is constructed by setting an edge between two TF if they share more target genes than expected by chance. All TF sharing at least one target gene were considered as potential co-regulators and tested using the same statistical selection (Fisher's test and multiple hypothesis testing correction). A similar approach was used in a recent study to identify Transcriptional Modules to describe the sets of TF involved in the same transcriptional programs [Fletcher et al., 2013].

The inferred pairs of co-regulators were compared to the protein interactions referenced by four studies : the FANTOM screen for combinatorial TF [Ravasi et al., 2010], the HIPPIE [Schaefer et al., 2012], HPRD [Prasad et al., 2009] and STRING [Franceschini et al., 2012] protein interaction databases. The enrichment of the inferred co-regulator networks in real protein-protein interaction is reported in table 2.

In order to further investigate the relevance of these predicted cooperative links between regulators, an evaluation of the predictive power of the inferred

	Network inference	Regulatory data integration	Co-regulator prediction	Genomic data integration	Transcription factor activity	Interactive network visualization	Differential analysis	availability
CLR	yes	no	no	no	no	no	no	Bioconductor (Minet)*
GENIE3	yes	no	no	no	no	no	no	R script
WGCNA	yes	no	no	yes	no (module activity)	no	yes	CRAN
RTN (ARACNE)	yes	no	no	yes	no	no	yes	Bioconductor
ROBNCA	no	yes	no	no	yes	no		matlab script
PLSgenomics	no	yes	no (infers meta-tf)	no	yes	no	no	CRAN
Bionet	no	no	no	no	no	no	yes	Bioconductor
Netbox	no	no	no	no	no	no	yes	python script
VAN	no	no	no	no	no	no	yes	R package
DEGraph	no	no	no	no	no	no	yes	Bioconductor
JActiveModule	no	no	no	no	no	yes	yes	Cytoscape
CorRegNet	yes	yes	yes	yes	yes	yes	yes	Bioconductor

Table 1: R packages and other tools for analyzing biological regulatory networks in the context of cancer genomics. CLR [Faith et al., 2007], GENIE3 [Huynh-Thu et al., 2010], WGCNA [Langfelder and Horvath, 2008], RTN [Fletcher et al., 2013], ARACNE [Margolin et al., 2006], ROBNCA [Noor et al., 2013], PLSgenomics [Boulesteix and Strimmer, 2005], BioNet [Beisser et al., 2010], Netbox [Cerami et al., 2010], VAN [Jayaswal et al., 2013], DEGraph [Jacob et al., 2010], JActivemodule [Ideker et al., 2002]. *CLR has several third-party implementations.

inferred co-regulators enrichment

	FANTOM	HIPPIE	HPRD	STRING
CoRegNet	2.43	2.6	2.75	3.75
GENIE3	1.63	1.83	1.74	2.83
WGCNA	1.5	1.55	1.6	2.62
CLR	1.51	1.69	1.59	2.17
ARACNE	1.44	1.49	1.5	1.8

Table 2: **Co-regulation enrichement in protein interaction.** Table of enrichment, computed as Odds Ratio, of Protein-Protein interactions found among inferred cooperative TF-TF. All enrichment are significant (Fisher's exact test $\alpha = 1\%$).

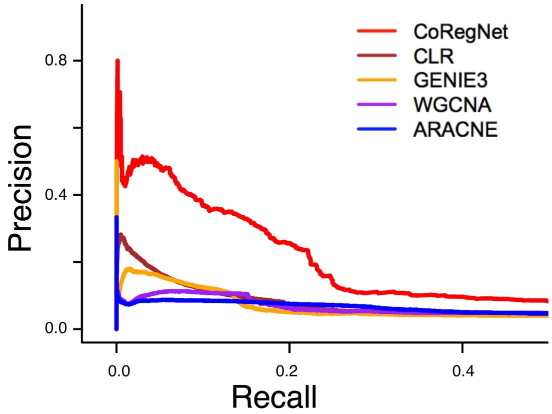

inferred co-regulators AUPR

	FANTOM	HIPPIE	HPRD	STRING
CoRegNet	1.54%	1.55%	1.54%	15.66%
GENIE3	0.78%	0.78%	0.78%	5.74%
WGCNA	0.83%	0.83%	0.83%	5.82%
CLR	0.80%	0.80%	0.80%	6.33%
ARACNE	0.77%	0.77%	0.77%	5.61%

Figure 1: **Precision-Recall analysis for the predictions of Protein-Protein interactions between co-regulators.** a. Precision-Recall curves computed using the predicted protein interaction in STRING as ground truth. Bottom table reports the Area Under Precision Recall curve (AUPR).

co-regulators was carried out. The objective here is to determine wether these cooperative links are able to predict PPI between two co-regulators. Therefore, in the context of a supervised binary classification, inferred pairs of co-

regulators were ordered by the number of shared target genes. This score was used to draw the Precision Recall curve which is shown in figure 1 using the STRING database [Franceschini et al., 2012] as ground truth. It is to be noted that STRING references experimentally identified protein interactions as well as predicted interactions based on several types of protein analysis such as phylogenic or literature mining. Therefore, STRING is here considered as referencing highly relevant functional relationships between proteins. These protein-level associations have been successfully used for biological predictions of operon for instance [Taboada et al., 2010]. This analysis was also done for the three other PPI ressources. However, as these ressources reference a much smaller number of PPI (10 to 20 times less than STRING), no visual representation of the Precision Recall curve is given here. Moreover, the AUPR, defined as the Area Under the Precision Recall curve, gives only little information using these sparse ressources (CoRegNet AUPR min: 1.54% max : 1.55% ; other methods min : 0.76% max : 0.83%).

Transcription Factor Activity

The CoRegNet influence is more robust to noise in the network

The Transcription Factor Activity (TFA) of all regulators was computed using the original inferred network. Noise was then added to the network by permuting an increasing portion (5%, 10% and 20%) of the target genes of each TF. This process was repeated 10 times resulting in 10 partially permuted network and 10 versions of noisy TFA for each levels of noise. The Pearson correlation of each regulator was computed between the TFA of the noisy and of the original networks. Figure 2 shows the distribution of Pearson's R^2 of the 10 noisy TF networks (resulting in $10 \times nTF$ correlation measures). Overall, the *influence* is much more resistant to noise in the prediction of regulatory interactions.

To further compare these methods, the original TFA of all TF in the network for which TF Binding Sites (TFBS) or public ChIP-seq data is available was correlated to the TFA computed using only the validated targets of each TF. The TFA of all regulators was computed using the original inferred network and on all samples of the dataset. Three networks were then derived from the original one by selecting only regulatory interactions present in one of the three datasets of regulatory evidences : ChEA and ENCODE for ChIP data and a collection of TFBS model. These three refined networks were used to compute a *validated* TFA with the three methods. Figure 3 shows the distribution of Pearson's correlation (R^2) for each TF between its activity measured with the original and in the *validated* network.

The CoRegNet influence accurately predicts real TF activation

Finally, these three methods were tested on a dataset in which the activation status of the TF PPARγ is known (data published in [Böck et al., 2014]). In essence, urothelial cells were cultivated with a PPARγ agonist (Roziglitazone)

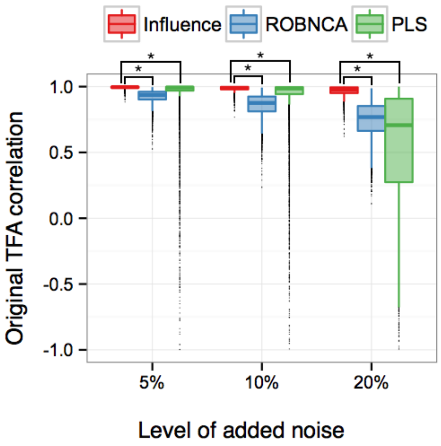

Table of mean correlation

Noise	Influence	ROBNCA	PLS
5%	0.99	0.92	0.93
10%	0.98	0.86	0.91
20%	0.97	0.75	0.54

Figure 2: **Robustness of TFA measures to network errors.** Distribution and mean of Pearson's correlation R^2 between the original TFA and the one computed with a partially permuted networks. * : equality rejected by two tail Student's test with $\alpha > 10^{-5}$

in combination with PD153035 to prevent an EGFR-dependent phosphorylation and inhibition of PPARγ [Varley et al., 2009, Böck et al., 2014]. The cells were sampled at various times after the activation of PPARγ resulting in a time series (6 hours, 24 hours, 3 days and 6 days). In this experimental setting, PPARγ exhibits null to weak activation at confluence (starting at day 3) in non-treated cells, a modest activation as soon as 6 hours and to reach full transcriptional activation at 24 hours and maintain this state in treated cells. Based on these transcriptomes, the activity of PPARγ was computed using the three tested methods, including the *influence* of the CoRegNet package. The results are shown in figure 4.

The *influence* measure is concordant with the expected state of PPARγ activity whereas the ROBNCA method does not detect PPARγ activation at 24 hours nor a small increase at confluence in non-treated cells. The PLS based measure of TFA shows less difference between the two type of cultures (treated and non-treated), especially at day 6.

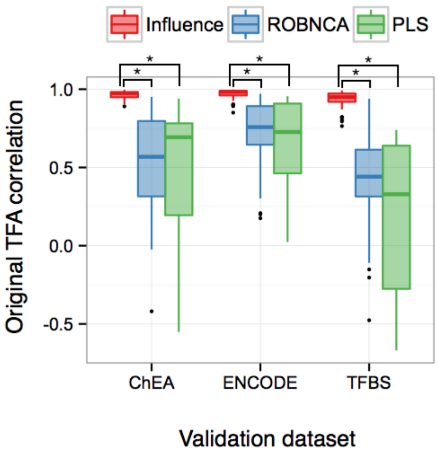

Table of mean correlation

Evidence	Influence	ROBNCA	PLS
ChEA	0.96	0.51	0.49
ENCODE	0.97	0.72	0.67
TFBS	0.93	0.42	0.19

Figure 3: **Validation of TFA measures.** Distribution and mean of the Pearson correlation R^2 between the original TFA and the one computed with a partially permuted network. $*$: equality rejected by two tail Student's test with $\alpha > 10^{-5}$.

2 Material and methods

Datasets

The transcriptomic data used throughout the study is a set of 179 transcriptomic profiles of Human bladder cancer samples and 4 normal bladder samples (the CIT dataset) [Rebouissou et al., 2014, Biton et al., 2014]. The cancer samples were also profiled for Copy Number Aberration (CNA) using CGH (comparative genome hybridization) chips.

The additional Human regulatory evidences originated from several sources. ChIP-seq or ChIP-on-chip data were directly downloaded from the ChEA2 database [Kou et al., 2013]. The Human ENCODE ChIP-seq data was recovered from the UCSC genome browser (Human hg19 February 2009 genome assembly) by selecting all narrow ChIP-seq peak (ENCODE chip V3) within -5000 bp to +2000 bp around a Transcription Start Site of a gene with a non-null Human genome organization Gene Nomenclature Committee (HGNC, genenames.org) symbol.

Human Transcription Factor Binding Sites (TFBS) models in the form of Position Weight Matrices (PWM) were recovered through the MotifDB R/Bioconductor package [Shannon, 2014] which references models from three different stud-

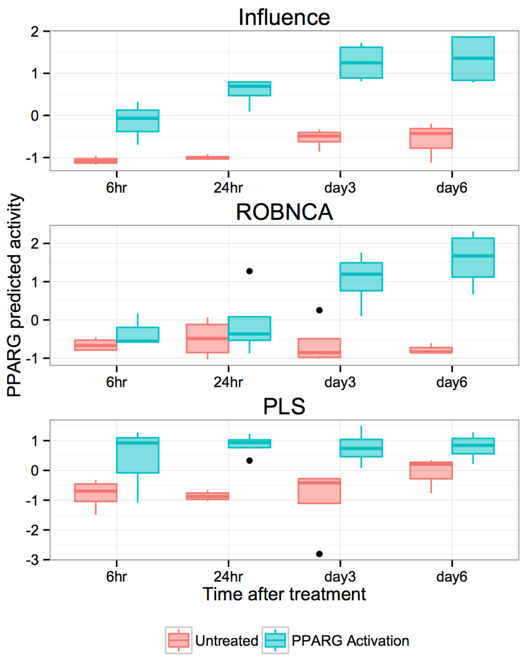

Figure 4: **Transcription Factor Activity of PPARγ.** Each time point correspond to 3 to 4 replicates. TFA for each methods was centered to 0 for visualization purpose.

ies [Portales-Casamar et al., 2009, Jolma et al., 2013, Xie et al., 2010]. This collection was complemented by the HOCOMOCO database of Human TFBS [Kulakovskiy et al., 2012]. When several models were available for the same Transcription Factor (TF), the PWM with the highest Information Content (in bits, see Stojnic and Diez [2014]) was kept. The promoter sequences (using the same coordinate that were used for the ENCODE ChIP-seq) were scanned for these sequences using the PWMEnrich R/Bioconductor package [Stojnic and Diez, 2014].

Human Protein-Protein Interactions (PPI) were downloaded from four different databases : HIPPIE [Schaefer et al., 2012], STRING [Franceschini et al., 2012], HPRD [Prasad et al., 2009] as well as from the FANTOM study of TF physical interaction through Mammalian Two Hybrid [Ravasi et al., 2010].

The large-scale regulatory network used throughout the following experiments is the result of the H-LICORN algorithm available in the COREGNET package applied with default parameters on the CIT bladder cancer dataset.

Co-regulatory network inference

The h-Licorn regulatory network inference algorithm implemented in CoRegNet requires a gene expression dataset and a set of TF. A list of Human transcription factors necessary to the construction of the regulatory network was compiled from the TRANSFAC database [Matys et al., 2006] and the FANTOM consortium [Ravasi et al., 2010] and is provided as an internal dataset of the package.

For each target gene g, *i.e.* non-TF coding genes, h-Licorn [Elati et al., 2007, Chebil et al., 2014] uses a discretized version of the transcriptomic data to identify a set of at least n candidate Gene Regulatory Networks GRN_1, GRN_2 $, \cdots, GRN_n$. For a given gene, g, a GRN is composed of a set of co-activators (A) and a set of co-inhibitors (I), $GRN = (A, I, g)$ in which A and I are sets of TF where both cannot be empty and are non-intersecting. For all extracted GRN, a linear model is used to estimate the expression of g as follow:

$$\hat{g} = \beta + \sum_{j=1}^{q+p} \alpha_j * r_j + \alpha_a \prod_{k=1}^{q} a_k + \alpha_i \prod_{l=1}^{p} i_l$$

with q the number of co-activators $q = |A|$, p the number of co-inhibitors $p = |I|$ and r_x, a_x and i_x the expression of a regulator with $r_i \in A \cup I$. $\hat{g}$ is an estimation of the continuous expression of g. In this model, the regulators are predictor variables and the expression of the target g is considered as the response. Interaction terms are added for co-activators and co-inhibitors to model the TF synergistic effect. In this setting, the adjusted coefficient of determination $\bar{R}^2$ is used to score each proposed GRN model.

Based on this only, the continuous data can be used to *refine* the original network by selecting for each gene g the GRN with the maximum $\bar{R}^2$.

In order to enrich large-scale regulatory networks using external regulatory interactions, the CoRegNet package implements functions introduced by the modENCODE consortium [Marbach et al., 2012b] and applies it to the selection of local GRN models.

In essence, the goal is to score each GRN (each interaction in the original method) using both the transcriptomic data and the integrated evidences to select the set of best GRN. Each GRN is scored by the inference method h-Licorn and by each of the integrated dataset. The number of interactions of a given GRN found in a given dataset of regulatory interactions is divided by the total number of predicted interactions ($|A|+|I|$). For cooperative evidences (TF-TF) such as protein interactions, all possible pairs of activators ($\frac{|A|\times(|A|-1)}{2}$) and all pairs of inhibitors ($\frac{|I|\times(|I|-1)}{2}$) are compared to the pairs of TF found in a given dataset. Finally, GRN are given this proportion of *validated* interactions as a score.

Following this, to each GRN is associated as many scores as their are integrated regulatory and cooperative datasets in addition to the network inference $\bar{R}^2$ score, all which range from 0 to 1. The original study [Marbach et al., 2012b] proposes two approaches to merge the scores, an unsupervised and a

supervised approach. While both are implemented in the CoRegNet package, the unsupervised approach was shown by the authors to have better performances [Marbach et al., 2012b]. It is simply an unweighted average of each of the scores. Finally, for each gene, the *GRN* with the maximum merged score is selected.

Cooperative regulators are defined as a set of regulators that are together necessary for the regulation of their target genes. To extract these set of co-regulators, all pairs of regulators which were found by h-Licorn to be co-activators or co-inhibitors of at least one gene, in at least one GRN, are considered as potential co-regulators in the studied context. Then, only those pairs which have a significant overlap of target genes using Fisher's exact test are predicted as co-regulators (with a 1% FDR control).

Transcription factor influence

The estimation of the influence of a TF requires a regulatory network structure defining for each TF a set of activated genes a and a set of inhibited genes i. The influence is then computed for a given sample using the average and standard deviation of the expression of these sets of genes (noted $\bar{X}_a$ and $\bar{X}_i$ for the mean and s_a^2 and s_i^2 for the standard deviation). For each TF and in each sample the influence is computed as follow :

$$\frac{\overline{X}_a - \overline{X}_i}{\sqrt{\frac{s_a^2}{|a|} + \frac{s_i^2}{|i|}}}$$

with $|a|$ and $|i|$ the number of activated and inhibited genes respectively.

Transcription Factor Activity (TFA) measurements were previously proposed. In order to evaluate the proposed measure of *influence*, two other linear-based methods of TFA predictions were used : an efficient implementation of the Network Component Analysis ROBNCA [Noor et al., 2013] and a Partial Least Square PLS-based method [Boulesteix and Strimmer, 2005]. These methods usually assume a trustful structure to estimate TFA [Liao et al., 2003]. However, network inference methods often predicts a substantial number of false regulatory interactions (*e.g.* low performance of the DREAM challengers in yeast, Marbach et al. [2012a]). Therefore TFA prediction methods should be able to cope with the noise, *i.e.* the prediction errors, in the networks. Therefore a first evaluation of the *influence* as a measure of TFA is its robustness to noise in the input network.

Integrative visualization of transcriptional activity

The visualization tool is based on a shiny application (shiny.rstudio.com), a web-page framework for R. The application has two pages, the main interactive analysis titled Co-regulation and visualization and the network Snapshot page. The Co-regulation page is divided in three parts (see figure 5) corresponding to a control panel, an interactive view of the co-regulator network and a plotting

panel to display network or TF-related data. The color of the nodes reflects the activity of the TF in the selected subtype as shown in figure 9 for two subtypes of bladder cancers.

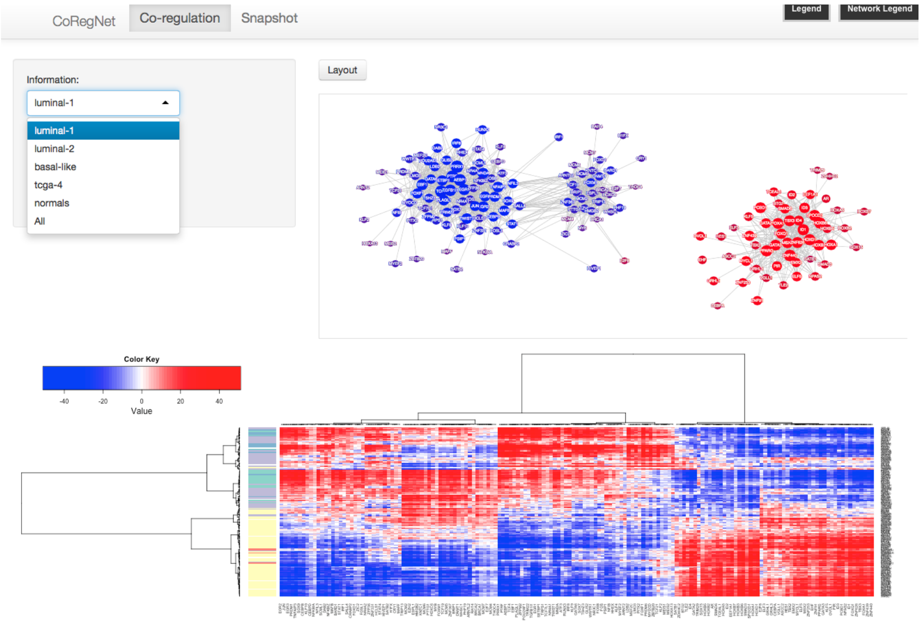

Figure 5: The visual application. Screenshot of the Shiny/R web application launched in CoRegNet. In the top left corner, a control panel lists the samples and sample subtypes to analyze, the number of minimum GRN to select significant cooperative interactions and an input to search for a particular TF in the network. In the right part, an interactive Cytoscape javascript widget display the network of co-regulators. The color of the nodes reflects the activity of the TF in the selected subtype, red, high activity; blue low activity. The bottom part of the page contains a plot reactive to action performs on the network.

When no nodes are selected in the Cytoscape widget, a heatmap of the TF influence is displayed. When several nodes are selected, the heatmap will contain only the influence of the selected TF. The selection of a single TF will display a multi-layer heatmap for each type of information given as an input to the application. An example is shown in figure 6.

Finally, when additional regulatory evidences were integrated in the network, the Cytoscape network will display these interactions in addition to the inferred co-regulation interactions as shown in figure 7. Regulatory evidences will be displayed as directed edges between TF while cooperative evidences will be shown as undirected edges.

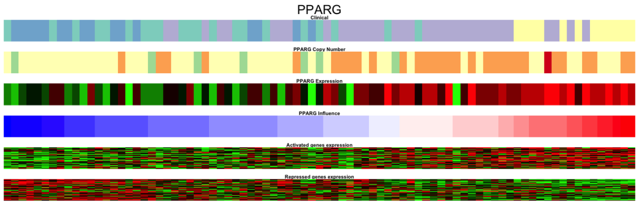

Figure 6: Local TF related heatmap. Expression is color coded from green to red (low to high) and the influence from blue to red (low to high). Heatmaps display one sample per column. The first heatmap color codes the sample classification. The second shows the Copy Number status of the select TF. The third and fourth show the expression and influence values of the selected TF. Finally, the fifth and sixth heatmap display the expression of the activated and repressed genes respectively.

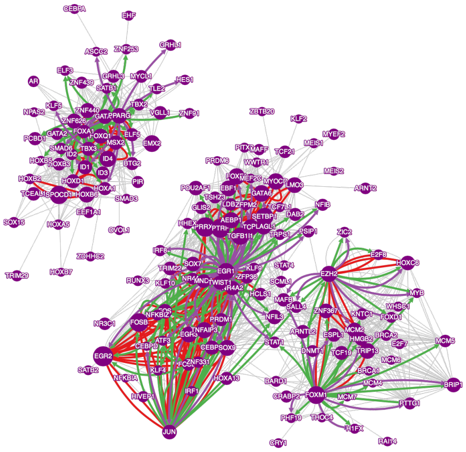

Figure 7: Multiple type of interactions between co-regulators inferred by CoRegNet. Grey : predicted cooperative interactions. Green : regulatory interactions from the ENCODE ChIP-seq data. Purple : regulatory interactions from the CHEA2 ChIP data. Red : protein interaction from the STRING database.

3 Case study

The gene expression data contains the expression of 18,901 genes in 183 samples. The unfiltered regulatory network inferred using the h-LICORN algorithm resulted in a regulatory network of 1004 TF and 9,486 target genes. The influence was computed in the same samples for TF with a sufficient number of target genes, at least 10 activated and 10 repressed genes, resulting in a matrix of the influence of 815 TF in the 183 samples. The samples were classified using the TCGA classification [Network, 2014] as in [Biton et al., 2014].

To identify driver TF, the effect of copy number gain on high influence was tested using Student's t-test to compare the influence of samples with gain to the other samples. Figure 8 shows a plot of the log p-value of this test in association with the mean influence of each TF in each subtype.

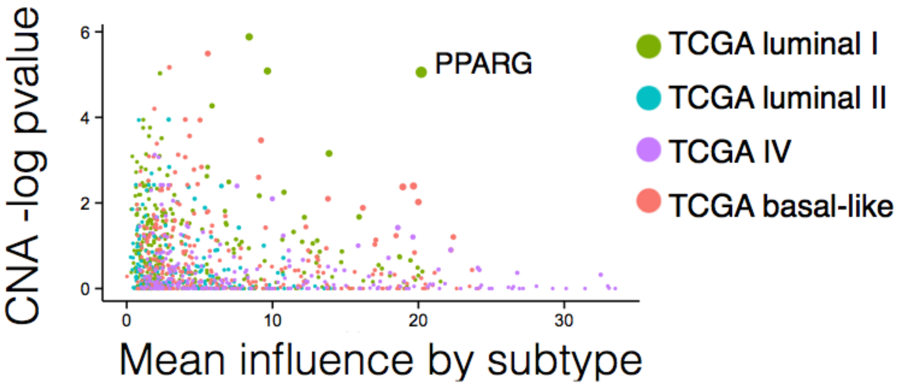

Figure 8: **Subtype influence and CNA.** Each point represents a TF in a given subtype distinguishable by its color. Green points correspond to the TCGA luminal 1 subgroup. Blue points correspond to the TCGA luminal 2 subgroup. Purple points correspond to the TCGA IV subgroup. Red points correspond to the TCGA basal-like subgroup (TCGA III).

PPARγ shows both a high influence in the TCGA Luminal 1 bladder cancer subtype and a high concordance between copy number gain and increase transcriptional activity. In line with recent findings [Choi et al., 2014, Biton et al., 2014], this result suggests PPARγ as a driver TF of the luminal subtype I, in which it is the third most active TF.

FOXA1 is a key effector of the activity of PPARγ in normal urothelial cells [Varley et al., 2009], which is responsible for the urothelial differentiation features observed in the luminal subtypes of bladder cancer. The most significant co-regulator of FOXA1 in the network was found to be PPARγ. Moreover, a known co-factor of FOXA1 in luminal breast cancer, GATA3 [Kong et al., 2011], was identified as the third most significant co-regulators of FOXA1.

References

Daniela Beisser, Gunnar W Klau, Thomas Dandekar, Tobias Müller, and Marcus T Dittrich. Bionet: an r-package for the functional analysis of biological networks. *Bioinformatics*, 26(8):1129–1130, 2010.

Anne Biton, Isabelle Bernard-Pierrot, Yinjun Lou, Clémentine Krucker, Elodie Chapeaublanc, Carlota Rubio-Pérez, Nuria López-Bigas, Aurélie Kamoun, Yann Neuzillet, Pierre Gestraud, et al. Independent component analysis uncovers the landscape of the bladder tumor transcriptome and reveals insights into luminal and basal subtypes. *Cell reports*, 9(4):1235–1245, 2014.

Matthias Böck, Jennifer Hinley, Constanze Schmitt, Tom Wahlicht, Stefan Kramer, and Jennifer Southgate. Developmental Biology. *Developmental Biology*, 386(2):321–330, February 2014.

Anne-Laure Boulesteix and Korbinian Strimmer. Predicting transcription factor activities from combined analysis of microarray and ChIP data: a partial least squares approach. *Theor Biol Med Model*, 2:23, 2005.

Ethan Cerami, Emek Demir, Nikolaus Schultz, Barry S Taylor, and Chris Sander. Automated network analysis identifies core pathways in glioblastoma. *PloS one*, 5(2):e8918, 2010.

Ines Chebil, Rémy Nicolle, Guillaume Santini, C´ eline Rouveirol, and Mohamed Elati. Hybrid Method Inference for the Construction of Cooperative Regulatory Network in Human. *NanoBioscience, IEEE Transactions on*, 13 (2):97–103, June 2014.

Woonyoung Choi, Sima Porten, Seungchan Kim, Daniel Willis, Elizabeth R Plimack, Jean Hoffman-Censits, Beat Roth, Tiewei Cheng, Mai Tran, I-Ling Lee, Jonathan Melquist, Jolanta Bondaruk, Tadeusz Majewski, Shizhen Zhang, Shanna Pretzsch, Keith Baggerly, Arlene Siefker-Radtke, Bogdan Czerniak, Colin P N Dinney, and David J McConkey. Identification of Distinct Basal and Luminal Subtypes of Muscle-Invasive Bladder Cancer with Different Sensitivities to Frontline Chemotherapy. *Cancer Cell*, 25(2):152–165, February 2014.

Mohamed Elati, Pierre Neuvial, Monique Bolotin-Fukuhara, Emmanuel Barillot, François Radvanyi, and C´ eline Rouveirol. LICORN: learning cooperative regulation networks from gene expression data. *Bioinformatics*, 23(18): 2407–2414, September 2007.

Jeremiah J Faith, Boris Hayete, Joshua T Thaden, Ilaria Mogno, Jamey Wierzbowski, Guillaume Cottarel, Simon Kasif, James J Collins, and Timothy S Gardner. Large-scale mapping and validation of escherichia coli transcriptional regulation from a compendium of expression profiles. *PLoS biology*, 5(1):e8, 2007.

Michael NC Fletcher, Mauro AA Castro, Xin Wang, Ines de Santiago, Martin O'Reilly, Suet-Feung Chin, Oscar M Rueda, Carlos Caldas, Bruce AJ Ponder, Florian Markowetz, et al. Master regulators of fgfr2 signalling and breast cancer risk. *Nature Communications*, 4, 2013.

Andrea Franceschini, Damian Szklarczyk, Sune Frankild, Michael Kuhn, Milan Simonovic, Alexander Roth, Jianyi Lin, Pablo Minguez, Peer Bork, Christian von Mering, et al. STRING v9.1: protein-protein interaction networks, with increased coverage and integration. *Nucleic Acids Research*, 41(D1):D808–D815, December 2012.

Vân Anh Huynh-Thu, Alexandre Irrthum, Louis Wehenkel, and Pierre Geurts. Inferring Regulatory Networks from Expression Data Using Tree-Based Methods. *PLoS ONE*, 5(9):e12776, September 2010.

Trey Ideker, Owen Ozier, Benno Schwikowski, and Andrew F Siegel. Discovering regulatory and signalling circuits in molecular interaction networks. *Bioinformatics*, 18(suppl 1):S233–S240, 2002.

Laurent Jacob, Pierre Neuvial, and Sandrine Dudoit. Gains in power from structured two-sample tests of means on graphs. *arXiv preprint arXiv:1009.5173*, 2010.

Vivek Jayaswal, Sarah-Jane Schramm, Graham J Mann, Marc R Wilkins, and Yee H Yang. Van: an r package for identifying biologically perturbed networks via differential variability analysis. *BMC research notes*, 6(1):430, 2013.

Arttu Jolma, Jian Yan, Thomas Whitington, Jarkko Toivonen, Kazuhiro R Nitta, Pasi Rastas, Ekaterina Morgunova, Martin Enge, Mikko Taipale, Gonghong Wei, Kimmo Palin, Juan M Vaquerizas, Renaud Vincentelli, Nicholas M Luscombe, Timothy R Hughes, Patrick Lemaire, Esko Ukkonen, Teemu Kivioja, and Jussi Taipale. DNA-Binding Specificitiesof Human Transcription Factors. *Cell*, 152(1-2):327–339, January 2013.

Say Li Kong, Guoliang Li, Siang Lin Loh, Wing-Kin Sung, and Edison T Liu. Cellular reprogramming by the conjoint action of ERα, FOXA1, and GATA3 to a ligand-inducible growth state. *Molecular Systems Biology*, 7: 1–14, August 2011.

Yan Kou, Edward Y Chen, Neil R Clark, Qiaonan Duan, Christopher M Tan, and Avi Ma ayan. ChEA2: Gene-Set Libraries from ChIP-X Experiments to Decode the Transcription Regulome. In ..., pages 416–430. Springer Berlin Heidelberg, Berlin, Heidelberg, 2013.

Ivan V Kulakovskiy, Yulia A Medvedeva, Ulf Schaefer, Artem S Kasianov, Ilya E Vorontsov, Vladimir B Bajic, and Vsevolod J Makeev. HOCOMOCO: a comprehensive collection of human transcription factor binding sites models. *Nucleic Acids Research*, 41(D1):D195–D202, December 2012.

Peter Langfelder and Steve Horvath. Wgcna: an r package for weighted correlation network analysis. *BMC bioinformatics*, 9(1):559, 2008.

James C Liao, Riccardo Boscolo, Young-Lyeol Yang, Linh My Tran, Chiara Sabatti, and Vwani P Roychowdhury. Network component analysis: reconstruction of regulatory signals in biological systems. *Proc. Natl. Acad. Sci. U.S.A.*, 100(26):15522, 2003.

Daniel Marbach, James C Costello, Robert Küffner, Nicole M Vega, Robert J Prill, Diogo M Camacho, Kyle R Allison, Andrej Aderhold, Kyle R Allison, Richard Bonneau, Diogo M Camacho, Yukun Chen, James J Collins, Francesca Cordero, James C Costello, Martin Crane, Frank Dondelinger, Mathias Drton, Roberto Esposito, Rina Foygel, Alberto de la Fuente, Jan Gertheiss, Pierre Geurts, Alex Greenfield, Marco Grzegorczyk, Anne-Claire HAURY, Benjamin Holmes, Torsten Hothorn, Dirk Husmeier, Vân Anh Huynh-Thu, Alexandre Irrthum, Manolis Kellis, Guy Karlebach, Robert Küffner, Sophie Lèbre, Vincenzo De Leo, Aviv Madar, Subramani Mani, Daniel Marbach, Fantine Mordelet, Harry Ostrer, Zhengyu Ouyang, Ravi Pandya, Tobias Petri, Andrea Pinna, Christopher S Poultney, Robert J Prill, Serena Rezny, Heather J Ruskin, Yvan Saeys, Ron Shamir, Alina Sîrbu, Mingzhou Song, Nicola Soranzo, Alexander Statnikov, Gustavo Stolovitzky, Nicci Vega, Paola Vera-Licona, Jean-Philippe Vert, Alessia Visconti, Haizhou Wang, Louis Wehenkel, Lukas Windhager, Yang Zhang, Ralf Zimmer, Manolis Kellis, James J Collins, and Gustavo Stolovitzky. Wisdom of crowds for robust gene network inference. *Nature Methods*, 9(8):796–804, July 2012a.

Daniel Marbach, Sushmita Roy, Ferhat Ay, Patrick E Meyer, Rogerio Candeias, Tamer Kahveci, Christopher A Bristow, and Manolis Kellis. Predictive regulatory models in Drosophila melanogaster by integrative inference of transcriptional networks. *Genome Research*, 22(7):1334–1349, July 2012b.

Adam A Margolin, Ilya Nemenman, Katia Basso, Chris Wiggins, Gustavo Stolovitzky, Riccardo Favera, and Andrea Califano. ARACNE: An Algorithm for the Reconstruction of Gene Regulatory Networks in a Mammalian Cellular Context. *BMC Bioinformatics*, 7(Suppl 1):S7, 2006.

Volker Matys, Olga V Kel-Margoulis, Ellen Fricke, Ines Liebich, Sigrid Land, A Barre-Dirrie, Ingmar Reuter, D Chekmenev, Mathias Krull, Klaus Hornischer, et al. TRANSFAC and its module TRANSCompel: transcriptional gene regulation in eukaryotes. *Nucleic Acids Research*, 34(Database issue): D108–10, January 2006.

The Cancer Genome Atlas Research Network. Comprehensive molecular characterization of urothelial bladder carcinoma. *Nature*, pages 1–8, January 2014.

Amina Noor, Aitzaz Ahmad, Erchin Serpedin, Mohamed Nounou, and Hazem Nounou. ROBNCA: robust network component analysis for recovering transcription factor activities. *Bioinformatics*, 29(19):2410–2418, September 2013.

Elodie Portales-Casamar, Supat Thongjuea, Andrew T Kwon, David Arenillas, Xiaobei Zhao, Eivind Valen, Dimas Yusuf, Boris Lenhard, Wyeth W Wasserman, and Albin Sandelin. JASPAR 2010: the greatly expanded open-access database of transcription factor binding profiles. *Nucleic Acids Research*, 38 (Database):D105–D110, December 2009.

TS Keshava Prasad, Renu Goel, Kumaran Kandasamy, Shivakumar Keerthikumar, Sameer Kumar, Suresh Mathivanan, Deepthi Telikicherla, Rajesh Raju, Beema Shafreen, Abhilash Venugopal, et al. Human protein reference database-2009 update. *Nucleic acids research*, 37(suppl 1):D767–D772, 2009.

Timothy Ravasi, Harukazu Suzuki, Carlo Vittorio Cannistraci, Shintaro Katayama, Vladimir B Bajic, Kai Tan, Altuna Akalin, Sebastian Schmeier, Mutsumi Kanamori-Katayama, Nicolas Bertin, Piero Carninci, Carsten O Daub, Alistair R R Forrest, Julian Gough, Sean Grimmond, Jung-Hoon Han, Takehiro Hashimoto, Winston Hide, Oliver Hofmann, Atanas Kamburov, Mandeep Kaur, Hideya Kawaji, Atsutaka Kubosaki, Timo Lassmann, Erik van Nimwegen, Cameron Ross MacPherson, Chihiro Ogawa, Aleksandar Radovanovic, Ariel Schwartz, Rohan D Teasdale, Jesper Tegnér, Boris Lenhard, Sarah A Teichmann, Takahiro Arakawa, Noriko Ninomiya, Kayoko Murakami, Michihira Tagami, Shiro Fukuda, Kengo Imamura, Chikatoshi Kai, Ryoko Ishihara, Yayoi Kitazume, Jun Kawai, David A Hume, Trey Ideker, and Yoshihide Hayashizaki. An Atlas of Combinatorial Transcriptional Regulation in Mouse and Man. *Cell*, 140(5):744–752, March 2010.

Sandra Rebouissou, Isabelle Bernard-Pierrot, Aurélien de Reyniès, May-Linda Lepage, Clémentine Krucker, Elodie Chapeaublanc, Aurélie Hérault, Aurélie Kamoun, Aurélie Caillault, Eric Letouzé, Nabila Elarouci, Yann Neuzillet, Yves Denoux, Vincent Molinié, Dimitri Vordos, Agnès Laplanche, Pascale Maillé, Pascale Soyeux, Karina Ofualuka, Fabien Reyal, Anne Biton, Mathilde Sibony, Xavier Paoletti, Jennifer Southgate, Simone Benhamou, Thierry Lebret, Yves Allory, and François Radvanyi. EGFR as a potential therapeutic target for a subset of muscle-invasive bladder cancers presenting a basal-like phenotype. *Science Translational Medicine*, 6(244):244ra91–244ra91, July 2014.

Martin H Schaefer, Jean-Fred Fontaine, Arunachalam Vinayagam, Pablo Porras, Erich E Wanker, and Miguel A Andrade-Navarro. HIPPIE: Integrating Protein Interaction Networks with Experiment Based Quality Scores. *PLoS ONE*, 7(2):e31826, February 2012.

Paul Shannon. Motifdb: An annotated collection of protein-dna binding sequence motifs, 2014.

Robert Stojnic and Diego Diez. Pwmenrich: Pwm enrichment analysis, 2014.

Blanca Taboada, Cristina Verde, and Enrique Merino. High accuracy operon prediction method based on STRING database scores. *Nucleic Acids Research*, 38(12):e130–e130, July 2010.

CL Varley, EJ Bacon, JC Holder, and J Southgate. Foxa1 and irf-1 intermediary transcriptional regulators of pparγ-induced urothelial cytodifferentiation. *Cell Death & Differentiation*, 16(1):103–114, 2009.

Zhi Xie, Shaohui Hu, Seth Blackshaw, Heng Zhu, and Jiang Qian. hPDI: a database of experimental human protein-DNA interactions. *Bioinformatics*, 26(2):287–289, January 2010.

TCGA Basal-like bladder cancer co-regulator network

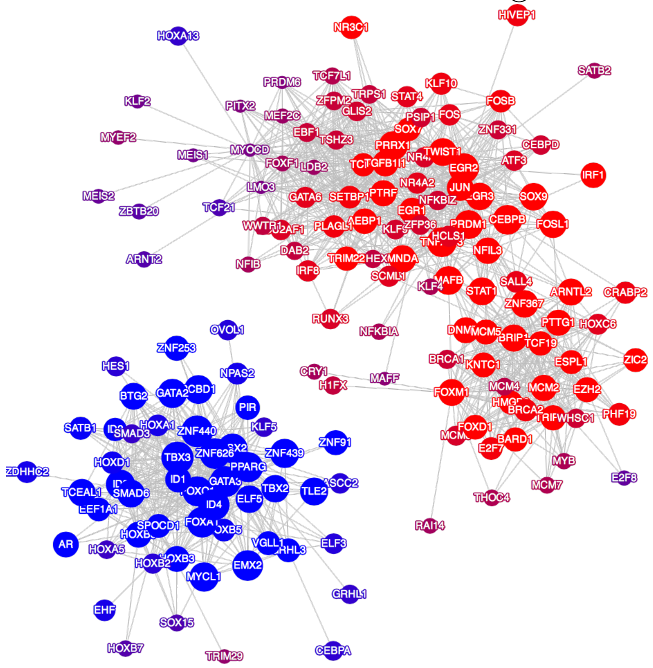

TCGA luminal 1 bladder cancer co-regulator network

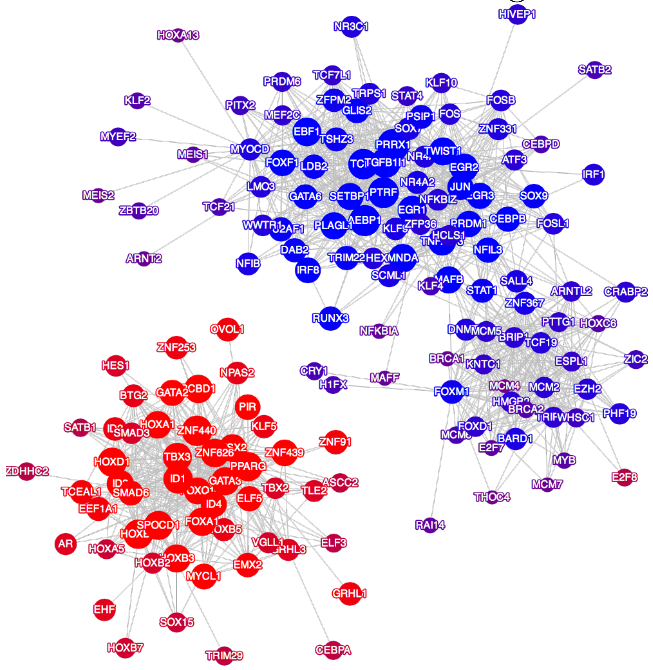

Figure 9: Subtype specific co-regulator network. The color of each TF/node is based on the mean influence in all samples of the subtype.
