# Supplementary material for: CoRegNet: reconstruction and integrated analysis of co-regulatory networks
Source: Bioinformatics. 2015 May 14;31(18):3066–8. doi: 10.1093/bioinformatics/btv305 (PMC4565029; doi:10.1093/bioinformatics/btv305)
Supplement: Supplementary Data [file supp_btv305_CoRegNet_1_2_0_tar.gz › CoRegNet/inst/doc/CoRegNet.html]

CoRegNet: Reconstruction and integrated analysis of Co-Regulatory Networks


# CoRegNet: Reconstruction and integrated analysis of Co-Regulatory Networks

#### *Remy Nicolle, Francois Radvanyi and Mohamed Elati*

#### *march 2015*

This Vignette accompanies the *CoRegNet* package. It can be used either to get some additional information about the methods or to get examples of the use of the functions. Feel free to ask any question to the package maintainer (remy.c.nicolle at gmail dot com).

# Introduction

The *CoRegNet* package aims at inferring a large scale transcription co-regulatory network from transcriptomic data and at integrating external data on gene regulation to infer and analyze transcriptional programs. The particularity of the network inference algorithm proposed in the package is to learn co-regulation network in which gene regulation is modeled by transcription factors acting in a cooperative manner to synergistically regulate target genes.

The package was used in a study of Bladder Cancer to identify the driver transcriptional programs from a set of 183 samples. Throughout this Vignette, a smaller version of the transcriptomic dataset is used to illustrate the use of the package.

```
        library(CoRegNet)
        data(CIT_BLCA_EXP,HumanTF,CIT_BLCA_Subgroup)
        dim(CIT_BLCA_EXP)
        #showing 6 first TF in the gene expression dataset
        head(intersect(rownames(CIT_BLCA_EXP),HumanTF))
```

# Quick user guide

Here are the main functionalities of *CoRegNet*

1. Reconstruct a large-scale regulatory network from gene expression data

   ```
       grn = hLICORN(CIT_BLCA_EXP, TFlist=HumanTF)
   ```
2. Infer transcription factor activity

   ```
       influence = regulatorInfluence(grn,CIT_BLCA_EXP)
   ```
3. Retrieve inferred co-coregulators

   ```
       coregs= coregulators(grn)
   ```
4. Analyze the network of cooperative regulators using an interactive display

   ```
       display(grn,CIT_BLCA_EXP,influence,clinicalData=CIT_BLCA_Subgroup)
   ```

# Construction of a large-scale co-regulatory network from gene expression data

The inference algorithm implemented in the package is a hybrid version of the LICORN algorithm. It is both based on a discrete and continuous version of the gene expression data. The reconstruction of the network involves 4 steps. First, the gene expression data is discretized. Second, all the potential sets of cooperative regulators are extracted using the *apriori* algorithm of frequent itemset mining. Third, the best combinations of co-activators and co-inhibitors are identified for each gene. Finally, a continuous model of regulation using a linear regression method with interaction terms is used to score the local gene regulatory networks of each gene.

The minimal input datasets is:

- a gene expression data.frame or matrix with unique column names and row names respectively containing sample and gene names
- a list of genes to be considered as Transcription factors (TF). The package contains a default list of Human TF either in the form of official gene symbol ( *data(HumanTF)* ) or EntrezGene IDs ( *data(HumanTF\_entrezgene)* ).

```
    # An example of how to infer a co-regulation network
    grn =hLICORN(CIT_BLCA_EXP, TFlist=HumanTF)
    print(grn)
```

The transcriptomic dataset is by default discretized using the *discretizeExpressionData* function. This function can be used to discretize gene expression data with or without a set of reference samples, such as healthy samples in the case of the study of a disease. In both cases, the expression of genes is centered either towards the mean expression of all samples when no reference samples are given or towards the mean of the set of reference samples. Note that the expression is not scaled to obtain a unitary standard deviation. Generally, even when reference samples are available, centering gene expression on the mean of all experiments performs well, especially on large datasets. The expression values are then set to +1 when it exceeds a given threshold or to -1 when it goes below a given threshold or to 0 otherwise. The choice of the threshold is either user defined or computed based on the overall distribution of the dataset.

Moreover, any discretized data can be used as an input, as long as the discretized matrix has similar dimensions and dimension names than the continuous expression data. For instance, a mixture of Gaussian can be fitted to each gene to identify samples in which a given gene is over-expressed, under-expressed or does not change.

```
    #Default discretization. 
    #Uses the standard deviation of the whole dataset to set a threshold.
    disc1=discretizeExpressionData(CIT_BLCA_EXP)
    table(disc1)
    boxplot(as.matrix(CIT_BLCA_EXP)~disc1)

    #Discretization with a hard threshold
    disc2=discretizeExpressionData(CIT_BLCA_EXP, threshold=1)
    table(disc2)
    boxplot(as.matrix(CIT_BLCA_EXP)~disc2)

    # more examples here
    help(discretizeExpressionData)
```

The overall inference process is highly parallelizable since it can be independently launched on each target gene and therefore can be divided in as many threads as there are genes to infer regulators on. By default the *hLICORN* function uses the *mclapply* function of the *parallel* package (default in R >= 3.0), which itself uses 2 cores by default.

```
# running only on the 200 first gene in the matrix for fast analysis
    # Choosing to divide in 4 threads whenever possible
    options("mc.cores"=4)
    grn =hLICORN(head(CIT_BLCA_EXP,200), TFlist=HumanTF)
    print(grn)
    options("mc.cores"=2)
    grn =hLICORN(head(CIT_BLCA_EXP,200), TFlist=HumanTF)
    print(grn)
```

Although *hLICORN* is an inference algorithm that is particularly efficient in identifying co-regulators, *e.g.* cooperative transcription factors, the package allows the input of a regulatory network inferred by other methods or defined by high-throughput biological experiments for the rest of the analysis.

# Refining the inferred regulatory networks

The second step of the analysis uses external knowledge to enrich the inferred regulatory network. Two types of external data can be used: regulatory information such as Transcription Factor Binding Sites (TFBS) or ChIP data to support TF to gene interactions and co-regulatory information such as protein-protein interaction to support cooperative TFs. These datasets can be easily integrated in a *coregnet* object using two functions: *addEvidences* and *addCooperativeEvidences*.

```
    # ChIP data from the CHEA database
    data(CHEA_sub)

    #ChIP data from the ENCODE project
    data(ENCODE_sub)

    # Protein protein interactions between TF from the HIPPIE database
    data(HIPPIE_sub)

    # Protein protein interactions between TF from the STRING database
    data(STRING_sub)

    enrichedGRN = addEvidences(grn,CHEA_sub,ENCODE_sub)
    enrichedGRN = addCooperativeEvidences(enrichedGRN,HIPPIE_sub,STRING_sub)
```

The *coregnet* object with added evidences contains a statistical analysis of the enrichment of the inferred interactions in the validated external evidences using a fisher exact test as exemplified below.

```
    print(enrichedGRN)
```

The added evidences can be used to refine the inferred network and select local gene regulatory networks. This is done based on the work of the *modEncode* consortium which proposed a method to integrate orthogonal datasets (transcriptome, epigenetic marks, binding sites, …) to learn a global network. Briefly, for each dataset, the method assigns one score to all possible interactions. The final score of each interaction merges each individual dataset score to select the best regulatory interactions. Here, instead of scoring and selecting single edges, the methods is extended to work with local regulatory networks (sets of co-activators and co-inhibitors of a given gene). For each dataset, a score is assigned to each local network by counting the number of validated interactions found in the local network normalized by the total number interactions. The score given by the network inference method, an adjusted R2 in the case of *hLICORN*, and each of the scores given by the external datasets are then merged. Two methods are proposed by the *modEncode* consortium to do so. The default is the unsupervised method, a simple unweighted mean, and was shown by the modENCODE consortium to give the best results. The supervised method uses a user defined reference dataset as a reference set of interactions. A Generalized Linear Model is then used to predict the presence of a reference interaction based on the score of the other datasets including the network inference score.

```
        # Default unsupervised refinement method
    refinedGRN = refine(enrichedGRN)
    print(refinedGRN)
    # Example of supervised refinement with the CHEA chip data
    refinedGRN = refine(enrichedGRN, integration="supervised",
            referenceEvidence="CHEA_sub")
    print(refinedGRN)
```

*N.B.*: It is possible to use the results of other network inference methods and integrate them into a *coregnet* network object as external regulatory evidences. However, the integration of co-regulatory evidences relevant when using networks containing cooperative regulators instead of simple TF to gene interaction pairs.

# Identification of active transcriptional programs

The goal of the *CoRegNet* package is to identify the sets of active cooperative TF in a given sample or set of samples. A measure of transcriptional activity was developed to estimate the level of activity of a given transcription regulator in a given sample. This measure, the *influence*, is the comparison of the expression of the activated and repressed genes of a TF in a transcriptional network. It is based on a measure of divergence (Welch’s t statistics) of these two sets of genes in one sample. Basically, if the genes activated by a TF are highly expressed while the repressed genes are under-expressed, the TF has a high influence. Using a coregulatory network encoded in a *coregnet*  
object and a matrix of gene expression, whether it is the data used for the inference or another one (cell lines transcriptomes for instance). The output is a matrix with the same number of columns (samples) and as many rows (TF) as TF with a sufficient number of targets (min 10 activated and 10 repressed) in the transcriptional network.

```
    CITinf =regulatorInfluence(grn,CIT_BLCA_EXP)
```

This new dataset of transcriptional influence can be used as a condensed view of the whole transcriptome dataset. Data mining and machine learning algorithms can be applied to it to identify clusters, predict sample classes or extract relevant features. More interestingly, it can be used to visualize the entire dataset with a much smaller number of features. The package proposes a visualization tool based on an interactive shiny webpage. The objective of the embedded visualization tool is to navigate through the transcriptional programs using the network of cooperative transcription factors and identify the specific transcriptional program of a sample or a set of samples defined by a subgroup of interest.

Preliminary to the analysis of transcriptional program is the identification of the network of co-regulators underlying these programs. The definition of a pair of cooperative TF or co-regulator is dependent on the method used to construct the network, that is, whether it contains simple pairs of regulation (TF -> Gene, inferred using the ARACNE algorithm for instance) or sets of cooperative TF regulating each gene ({TF1, TF2, …} -> Gene, inferred using the *hLICORN* algorithm embedded in the package). From the pair regulatory network, all combination of TF sharing at least one target gene is considered as a potential pair of co-regulator. From the cooperative network, these are defined by all pairs of TF which were inferred to be cooperative in the regulation of at least one target gene. Then, the significance of these pairs is tested using fisher’s exact test to assess whether these pairs of TF share more target genes than expected by chance (Benjamini-Hochberg multi test correction is applied). The final co-regulation network is defined by an edge between all pairs of significant TF (FDR 1%).

```
    # Coregulators of a hLICORN  inferred network
    head(coregulators(grn))
```

In order to exemplify the use of the proposed analysis tool, additional data are available through the package: the classification of our samples (based on the TCGA classification) and the copy number status of the transcription factor in the CIT cohort.

```
    data(CIT_BLCA_CNV)
    data(CIT_BLCA_Subgroup)
```

The minimum requirement to launch the visualization tool is a *coregnet* network object and a transcriptomic dataset. The influence dataset can also be added to accelerate the initialization of the webpage but is otherwise recomputed and is therefore optional.

```
    display(grn,expressionData=CIT_BLCA_EXP,TFA=CITinf)
```

The top left part of the shiny application is used to change several parameters of the application.

The first selection input is usable only if a sample classification is available. When a particular subgroup is selected, the nodes representing TF will adapt their color based on their mean activity level in the selected set of samples. If alteration data is available, the pie chart inside each node will by modified to show the proportion of each copy number status in the selected set of samples.

The second input specifies an integer used as a threshold to select pairs of TF which share a minimum number of target genes (local regulatory network for *hLICORN* inferred network). This parameter controls the number of TFs and pairs of TFs displayed.

The third input can be used to search a TF in the network.

The layout button can be used to change the layout of the network. This can be a long process for large networks.

The bottom panel is used to display specific plots. A heatmap of TF influence is displayed by default. If only a subset of TF is selected in the cytoscape panel, the heatmap will only display the influence of the selected TF. Selecting a single TF will display data specific to that TF which is dependent on the data used as input. Each of the data is represented as an array of color ordered by the activity of the selected TF. The plotted data includes the expression of the TF, the activity of the TF and the expression of the activated and the repressed samples.

The visualization tool can also handle alteration data, sample classification and evidences of regulation or co-regulation. Several examples are shown below.

```
    # Visualizing additional regulatory or co-regulatory evidences in the network
    display(enrichedGRN,expressionData=CIT_BLCA_EXP,TFA=CITinf)

    # Visualizing sample classification using a named factor
    display(grn,expressionData=CIT_BLCA_EXP,TFA=CITinf,clinicalData=CIT_BLCA_Subgroup)

    # Visualizing copy number alteration of regulators
    data(CIT_BLCA_CNV)
    display(grn,expressionData=CIT_BLCA_EXP,TFA=CITinf,clinicalData=CIT_BLCA_Subgroup,alterationData=CIT_BLCA_CNV)
```

*N.B.*: the Cytoscape network snapshot is experimental and usually is one snapshot late. Usually going back and forth to the snapshot tab once or twice should do the trick to have the latest snapshot. Hopefully this will be corrected by the Cytoscape.JS team.

---

# Snapshots and examples

The following contains snapshots of the shiny interactive user interface for the visualization of a co-regulatory network driving bladder cancer.

The Co-regulation page is divided in three parts (see figure 1). In the top left corner, a control panel lists the samples and samples subtype to analyze, the number of minimum GRN to select significant cooperative interactions and an input to search for a particular TF in the network. In the right part, an interactive Cytoscape javascript widget displays the network of co-regulators. The color of the nodes reflects the activity of the TF in the selected subtype as shown in figures 2 and 3 for two types of bladder cancers. The bottom part of the page contains a plot reactive to action performs on the network.


*Figure 1: View of the shiny Web page*

## Subtype specific co-regulator network. The color of each TF/node is based on the mean influence in all samples of the subtype

*Figure 2: Basal-like bladder cancer co-regulator network*
*Figure 3: Luminal-like bladder cancer co-regulator network*


---

When no nodes are selected in the Cytoscape widget, a heatmap of the TF influence is displayed (as in figure 1). When several nodes are selected, the heatmap will contain only the influence of the selected TF. The selection of a single TF will display a multi-layer heatmap for each type of information given as an input to the application. An example is shown in figure 4. The first heatmap color-codes the sample classification. The second shows the Copy Number status of the select TF. The third and fourth show the influence and expression values of the selected TF. Finally, the fifth and sixth heatmap display the expression of the activated and repressed genes respectively.

*Figure 4: Local TF related heatmap.* Expression is color coded from green to red (low to high) and the influence from blue to red (low to high). Heatmaps display one sample per column.


---

Finally, when additional regulatory evidences were integrated in the network, the Cytoscape network will display these interactions in addition to the inferred co-regulation interactions as shown in figure 5. Regulatory evidences will be displayed as directed edges between TF while cooperative evidences will be shown as undirected edges.

*Figure 5: Multiple types of interactions.* Grey: predicted cooperative interactions. Green: regulatory interactions from the ENCODE ChIP-seq data. Purple: regulatory interactions from the CHEA2 ChIP data. Red: protein interaction from the STRING database.
