# Supplementary material for: CoRegNet: reconstruction and integrated analysis of co-regulatory networks
Source: Bioinformatics. 2015 May 14;31(18):3066–8. doi: 10.1093/bioinformatics/btv305 (PMC4565029; doi:10.1093/bioinformatics/btv305)
Supplement: Supplementary Data [file supp_btv305_CoRegNet_1_2_0_tar.gz › CoRegNet/inst/www/js/jquery-slick/index.html]

jQuery Slick Plugin v 2.1


## jQuery Slick Plugin v 2.1 - Visit Plugin Site

Toggle Tabs

Close Tabs

Open Tabs

Dicat velit doctus cu his, te iriure feugait pri, semper quaestio id vel. Ius assum aliquid civibus eu. Comprehensam conclusionemque ne qui, vim ad malis partiendo. No illum lorem perfecto ius, et eos quas iusto.

Soleat malorum sed ei, ex mei sint sale mandamus, probo noluisse prodesset te nam. Mentitum volutpat dissentias cu eam, vim minim patrioque ea. Meis utroque inimicus ne mel. Epicurei splendide suscipiantur ius ut, kasd graece commune sed ei. Vis deseruisse intellegam ne, has probo ornatus et. No dicat etiam ius, dicit tantas ut sea.

- Home
- About
- Terms & Conditions
- Contacts

Soleat malorum sed ei, ex mei sint sale mandamus, probo noluisse prodesset te nam. Mentitum volutpat dissentias cu eam, vim minim patrioque ea. Meis utroque inimicus ne mel. Epicurei splendide suscipiantur ius ut, kasd graece commune sed ei. Vis deseruisse intellegam ne, has probo ornatus et. No dicat etiam ius, dicit tantas ut sea.

Eos ex summo facilisi iudicabit. Dico dolores delicatissimi eam eu. Quodsi definiebas nam ex. His ea ponderum ocurreret tincidunt, his eu nulla democritum temporibus. Possim timeam philosophia est ut, pro iracundia aliquando ne. Virtute reprimique eam ne, cu quo alii facer partiendo, nam eu natum mucius.

Soleat malorum sed ei, ex mei sint sale mandamus, probo noluisse prodesset te nam. Mentitum volutpat dissentias cu eam, vim minim patrioque ea. Meis utroque inimicus ne mel. Epicurei splendide suscipiantur ius ut, kasd graece commune sed ei. Vis deseruisse intellegam ne, has probo ornatus et. No dicat etiam ius, dicit tantas ut sea.

Eos ex summo facilisi iudicabit. Dico dolores delicatissimi eam eu. Quodsi definiebas nam ex. His ea ponderum ocurreret tincidunt, his eu nulla democritum temporibus. Possim timeam philosophia est ut, pro iracundia aliquando ne. Virtute reprimique eam ne, cu quo alii facer partiendo, nam eu natum mucius.

- Home
- About
- Terms & Conditions
- Contacts

- Home
- About
- Terms & Conditions
- Contacts
