# Supplementary figures and images for: CoRegNet: reconstruction and integrated analysis of co-regulatory networks
Source: Bioinformatics. 2015 May 14;31(18):3066–8. doi: 10.1093/bioinformatics/btv305 (PMC4565029; doi:10.1093/bioinformatics/btv305)

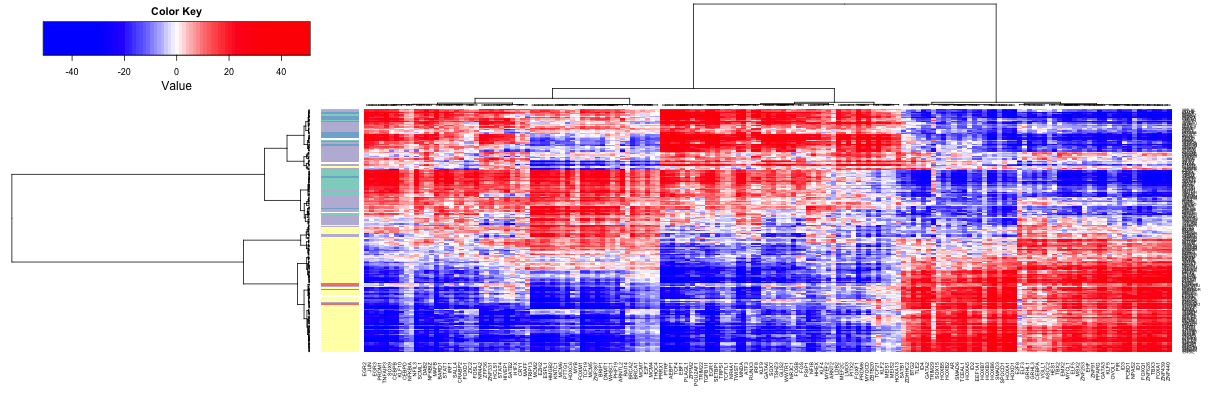

Supplement: Supplementary Data [file supp_btv305_CoRegNet_1_2_0_tar.gz › CoRegNet/vignettes/fig/InfluenceHeatmap.png]

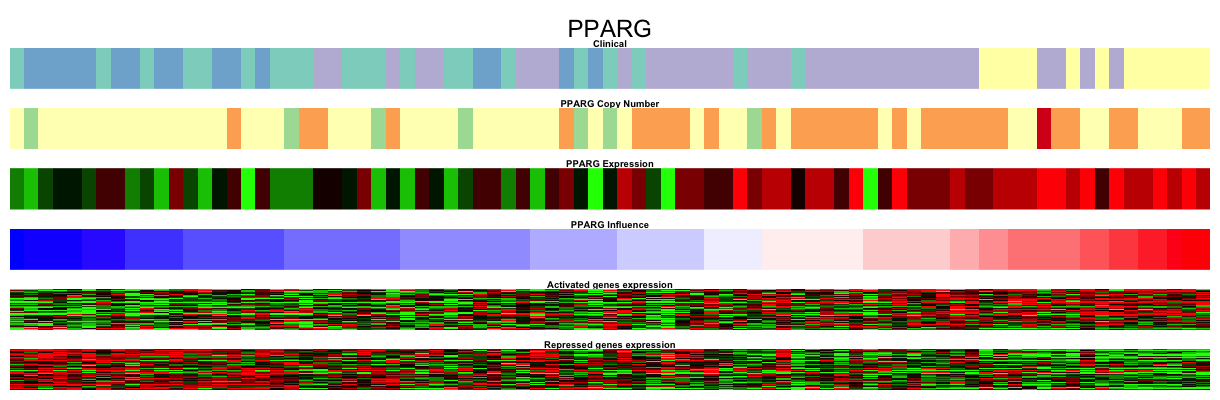

Supplement: Supplementary Data [file supp_btv305_CoRegNet_1_2_0_tar.gz › CoRegNet/vignettes/fig/PPARGLocalView.png]

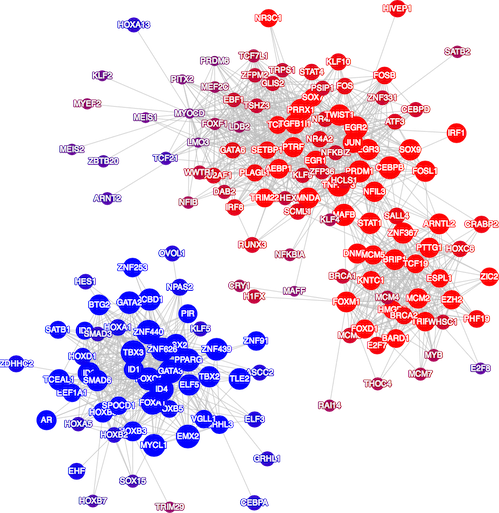

Supplement: Supplementary Data [file supp_btv305_CoRegNet_1_2_0_tar.gz › CoRegNet/vignettes/fig/basal1.png]

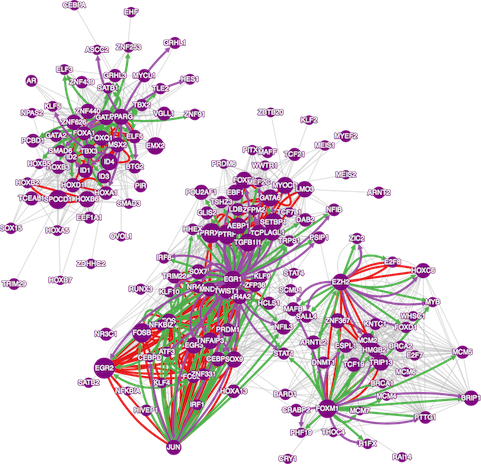

Supplement: Supplementary Data [file supp_btv305_CoRegNet_1_2_0_tar.gz › CoRegNet/vignettes/fig/coregnetWithEvidence.png]

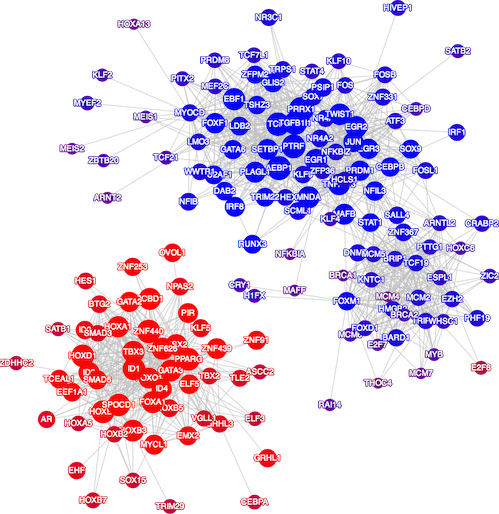

Supplement: Supplementary Data [file supp_btv305_CoRegNet_1_2_0_tar.gz › CoRegNet/vignettes/fig/luminal1.png]

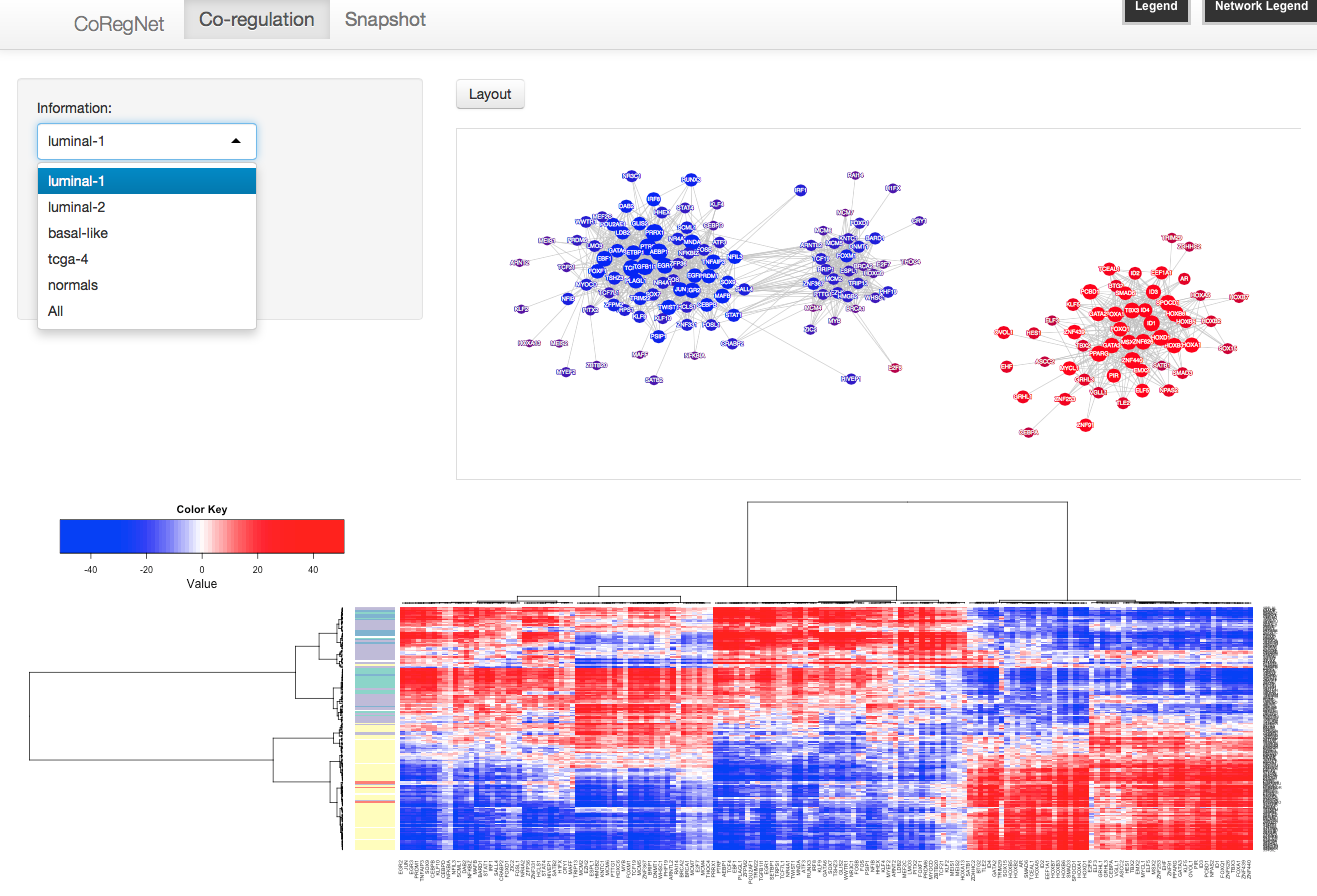

Supplement: Supplementary Data [file supp_btv305_CoRegNet_1_2_0_tar.gz › CoRegNet/vignettes/fig/wholeApp.png]
